# Designing a Virtual Hospital-at-Home Intervention for Patients with Infectious Diseases: A Data-Driven Approach

**DOI:** 10.3390/jcm13040977

**Published:** 2024-02-08

**Authors:** Harriët M. R. van Goor, Titus A. P. de Hond, Kim van Loon, Martine J. M. Breteler, Cor J. Kalkman, Karin A. H. Kaasjager

**Affiliations:** 1 Department of Internal Medicine, University Medical Centre Utrecht, 3584 CX Utrecht, The Netherlands; 2 Department of Anesthesiology, University Medical Centre Utrecht, 3584 CX Utrecht, The Netherlands; 3 Department of Digital Health, University Medical Centre Utrecht, 3584 CX Utrecht, The Netherlands; 4 Julius Centre for Health Sciences and Primary Care, University Medical Centre Utrecht, 3584 CX Utrecht, The Netherlands

**Keywords:** telemedicine, hospital-at-home care, infectious diseases

## Abstract

Background: Virtual hospital-at-home care might be an alternative to standard hospital care for patients with infectious diseases. In this study, we explore the potential for virtual hospital-at-home care and a potential design for this population. Methods: This was a retrospective cohort study of internal medicine patients suspected of infectious diseases, admitted between 1 January and 31 December 2019. We collected information on delivered care during emergency department visits, the first 24 h, between 24 and 72 h, and after 72 h of admission. Care components that could be delivered at home were combined into care packages, and the potential number of eligible patients per package was described. The most feasible package was described in detail. Results: 763 patients were included, mostly referred for general internal medicine (35%), and the most common diagnosis was lower respiratory tract infection (27%). The most frequently administered care components were laboratory tests, non-oral medication, and intercollegiate consultation. With a combination of telemonitoring, video consultation, non-oral medication administration, laboratory tests, oxygen therapy, and radiological diagnostics, 48% of patients were eligible for hospital-at-home care, with 35% already eligible directly after emergency department visits. Conclusion: While the potential for virtual hospital-at-home care is high, it depends greatly on which care can be arranged.

## 1. Introduction

The aim of hospital-at-home care is to provide hospital-level care in the patient’s home in a situation that would otherwise require hospital admission [1]. Besides creating a more positive healing environment for the patient [2], hospital-at-home interventions might reduce costs and resources [3]. It might also be a favorable alternative to hospitalization for frail elderly patients with a high risk of hospital complications, such as delirium [4]. Hospital-at-home has been used for decades for various populations and in a wide range of modalities [3]. Most of these interventions include in-person visits by hospital staff (nurses and/or physicians) and still require considerable (human) resources. An alternative for in-person care could be telemedicine, where some of—or potentially all—home visits are substituted with virtual visits and the telemonitoring of vital signs. This type of care has previously proven to be feasible and satisfactory [5,6], appears to be safe [5,6,7], and might reduce costs [8].

During the COVID-19 pandemic, the need for telemedicine options became more urgent, not only to handle high patient loads but also to reduce the amount of contact between caregivers and contagious patients. Telemedicine-based hospital-at-home interventions to avoid admission or facilitate early discharge were implemented in multiple countries for patients with COVID-19 [9,10,11,12,13,14]. Building on the success of these interventions, hospitals are now looking to continue and expand the availability of telemedicine-based hospital-at-home to a broader patient population [15]. However, the required facilities for telemedicine for COVID-19 patients are relatively simple, with oxygen therapy, dexamethasone, and vital signs monitoring being the cornerstones of therapy. The care that needs to be organized for other infectious diseases might be more elaborate, e.g., the need for intravenous antibiotics. For both currently known infectious diseases and possible future new epidemics, telemedicine designs might have to be adjusted. Furthermore, not all patients appear to be suitable candidates for telemedicine-based hospital-at-home interventions [6,9,11]. Optimal patient selection is thus necessary and might improve the effectiveness of hospital-at-home care.

In this study, we describe the characteristics of patients who are admitted to the internal medicine ward with a suspected infection. We aimed to gain insight into the components of hospital care these patients receive during hospitalization, the timing of received care, and whether differences in received care could be based on certain patient-, disease-, or admission characteristics. Based on this information, we suggest a design for a telemedicine-based hospital-at-home intervention for patients with suspected or diagnosed infectious diseases. Lastly, we explore factors that might indicate eligibility for hospital-at-home care.

## 2. Materials and Methods

We conducted an exploratory, retrospective, single-center cohort study, as part of the SePsis in ACutely ill patients in the Emergency room (SPACE) study [16]. SPACE is an ongoing observational cohort study; the database includes all patients with suspected infection who have presented at the emergency department (ED) of the tertiary hospital University Medical Centre Utrecht, Utrecht, the Netherlands, since September 2016. The ethical review of this study was waived by the MERC Utrecht (16-594, 20 September 2016). Considering the retrospective nature and high volume of the study, individual informed consent was deemed unnecessary by the institutional research board.

### 2.1. Study Population and Data Collection

For this study, only patients from the SPACE cohort who were admitted in the year 2019 were included. We chose this year since it is the most recent year that is not influenced by the COVID-19 pandemic, but since it is a full year, it does include all seasonal fluctuations. Adult patients who presented at the ED for the internal medicine department (or subspecialties) who were suspected to suffer from an infection and who were admitted to the hospital were included. Re-admissions were recorded as new cases, unless the re-admission occurred within 30 days, in which case only the first admission was included. Data of the included patients were collected from the electronic health record into case report forms. We collected data on patient characteristics manually, including age, sex, and medical history. The Modified Early Warning Score (MEWS) at ED admission, diagnosis, and treatment at the ED were also manually included in the case report forms. Additional information was collected using text mining of the electronic health record. Of the ED visit and subsequent admission, we collected timestamped data on performed diagnostic tests (laboratory tests and radiological diagnostic), consultation by different specialties, treatments administered (medication, intravenous fluid administration, oxygen administration, central intravenous catheter placement, urine catheter placement, feeding tube placement, and high care interventions such as surgery, scopic and radiologic interventions, and electrical cardioversion), available MEWS during admission, and assistance in activities of daily living (ADL) by hospital staff. ADL assistance was assessed by either help in feeding, bathing, or going to the toilet. No data could be obtained for assistance in ADL at the ED, and only the MEWS at admission was known for the ED. The MEWS during admission had a significant number of missing values (24.1%) which we chose not to impute since missingness was not at random.

### 2.2. Assumptions

We divided each admission into four stages: the ED visit, the first 24 h of admission to the hospital ward, between 24 and 72 h of admission, and the remainder of the admission. These stages were chosen since they represent important moments at which remote hospital care could be initiated: directly following ED presentation; after 24 h of admission; when hospital care has been initiated; or after 72 h of hospital treatment, when it is expected that the initiated therapy will have shown effect. Furthermore, in consultation with the ‘hospital-at-home’ program manager of the hospital, we determined which parts of hospital care were feasible and appropriate to offer at home. Rapid response team (RRT) consultation; intensive care unit (ICU) admission; high care interventions such as surgery, electrical cardioversion, and endoscopic procedures; and oxygen therapy with flows above 5 L/min were determined to be care components that can only be offered in hospital; all other components of care that were noted could theoretically be organized at home. Since the possibilities of performing radiological imaging at the patient’s home vary highly among regions, we assumed that all radiologic diagnostics had to be performed in the outpatient clinic. For a missing MEWS during admission, we assumed it was not above the threshold for action in the hospital’s protocol since a high MEWS is more likely to be recorded than a low MEWS. The implications of these assumptions will be outlined in the discussion of this study.

### 2.3. Descriptive Analysis

Using the collected data, we first described the patient characteristics of the entire cohort during the four stages of admission. We used this information to determine whether obvious subgroups could be distinguished, e.g., based on age or admission diagnosis. Secondly, we calculated the percentage of patients who received a certain component of hospital care during a specific stage of hospital admission, both for the entire cohort and for the admission diagnosis groups that were most common. We used this information to establish: 1. which components of hospital care were most frequent; 2. at which stage of hospital admission these components were administered; and 3. whether differences existed in the percentage of people receiving certain hospital care between subgroups. Next, for every patient, we established whether they had received hospital care that can only be offered in the hospital. This information was used to determine the number of patients that could theoretically be discharged after each stage (directly after ED presentation, after 24 h, after 72 h) and receive the remaining hospital care at home.

Initially, the goal was to see which combination of care components occurred most often. The total number of possible combinations of hospital care components (>150), however, was too high for categorization based on the combinations of received care. Instead, we decided to group the components into ‘packages’ of hospital care that could be offered at home, with increasingly more components and/or labor-intensiveness. The selection of these packages was based on the occurrence of the hospital care components (e.g., laboratory tests were very common and therefore included in most packages); the ease with which a particular component could be organized at home (e.g., a patient could administer his/her own subcutaneous medication after a short training, whereas a certified nurse and equipment are needed for intravenous administration); and the need for transportation (oxygen therapy can be administered at home, but radiology tests need to be performed within a care facility). Additionally, we added a package of hospital-at-home care with telemonitoring previously described by Summerfelt et al. [6], which has already been successfully implemented. For each care package, we determined the number of patients that could receive hospital-at-home care after each stage of admission. We selected the package that was deemed most efficient—which was defined as the most patients at home with the least complex and least amount of care components—to describe in more detail.

### 2.4. Statistical Analysis

As the final part of the exploration, we used a multivariate binary logistic model to determine factors that could predict whether a patient would be able to receive hospital-at-home care after each stage of admission. Clinically relevant variables were chosen and eliminated using backward selection. We constructed four models: one for the prediction of ‘being able to go home with the selected package’ at any point in time, and one for going home after each stage, respectively. A *p*-value < 0.05 was considered statistically significant. All analyses, both descriptive and statistical, were performed using SPSS version 26.0 (IBM Corp., IBM SPSS Statistics for Windows, Armonk, NY, USA).

## 3. Results

The cohort consisted of 763 admitted patients (Table 1). Patients were admitted mostly to the general internal medicine ward (35%), followed by oncology (21%) and nephrology (16%). The most common admission diagnoses were lower respiratory tract infection (LRTI) (27%), urinary tract infection (UTI) (19%), and gastrointestinal infection (GI) (18%). The most frequently occurring components of hospital care were laboratory tests, intravenous/other invasive (IV) and intradermal/subcutaneous/intramuscular (ID/SC/IM) medication administration, and intercollegiate consultation (Table 2). Intercollegiate consultation occurred most often with a colleague in internal medicine (12.1%), followed by geriatric medicine (10.8%), pulmonology (10.5%), cardiology (9.7%) and neurology (8.8%). The four most common components were also most common for patients with LRTIs, UTIs, or GIs. Patients with LRTIs received more other components of care compared with the overall cohort. Patients with UTIs more often needed a urine catheter (Supplemental Tables S1–S3). Radiologic diagnostics, especially X-ray or ultrasound, were most commonly performed at the ED (79% of all patients), and less during admission.

**Figure 1:**
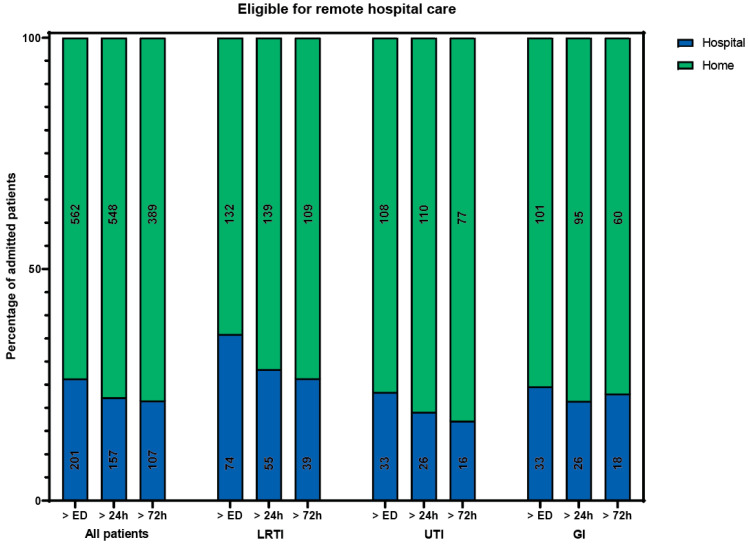
Percentage of patients eligible (in green) for remote hospital care if all components were available at home, besides rapid response team consultation, intensive care admission, high care intervention, or oxygen therapy > 5 L/min. Number of patients in a bar is presented in the bar. ED: emergency department. LRTI: lower respiratory tract infection. UTI: urinary tract infection. GI: gastrointestinal infection.

Of all included patients, 133 (17%) received care that could not have been delivered at home during all periods. The remaining 630 patients could theoretically have received hospital-at-home care at some point during admission, provided that all care components can be delivered at home. A total of 562 patients (74%) could, in theory, have received hospital-at-home care immediately following their ED visit (Figure 1). The admission specialties and diagnoses for patients in this group were the same as for the entire cohort. The median hospital length of stay (LOS) was 4 days (IQR 2–7). The most occurring components of hospital care (laboratory tests, intercollegiate consultation, IV and ID/SC/IM medication) were also the most common components in this group.

### 3.1. Analysis of Care Packages

**Figure 2:**
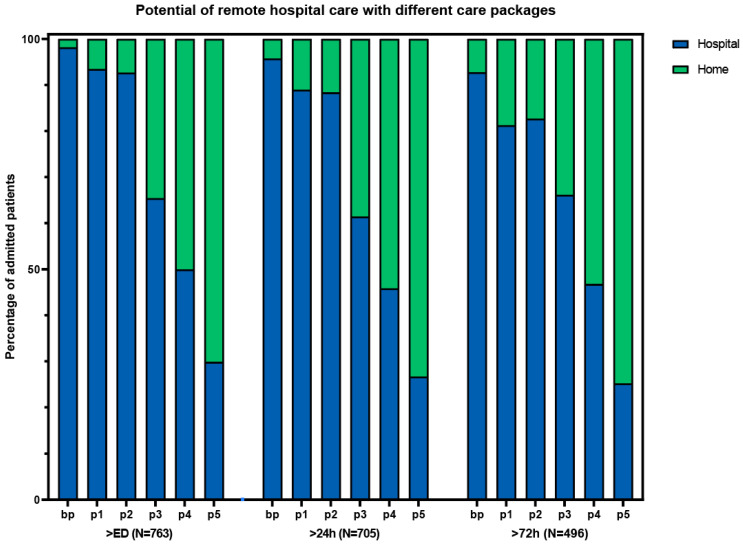
Potential of remote hospital care with different care packages. ED: emergency department. bp: basic package. p1: package 1 (bp + laboratory tests + ADL assistance + physiotherapy). p2: package 2 (bp + laboratory tests + radiology tests + oxygen). p3: package 3 (p2 + IV medication). p4: package 4 (p3 + ADL + physiotherapy). p5: package 5 (p4 + urine catheter OR central venous catheter OR feeding tube).

Based on the results and complexity of different components, we composed different packages of care (Table 3). Package 3 had a high number of patients who could receive care at home, with relatively few complex intervention components (Figure 2). This package consisted of the base package plus laboratory tests, IV medication, oxygen therapy, and the possibility of receiving radiology at home or in an outpatient setting. With this package, 362 (48%) of the patients could have gone home with hospital care at some point during admission. Packages without the option for IV medication (basic package and package 1) resulted in considerably fewer patients that would be eligible for hospital-at-home. The addition of ADL and physiotherapy to package 3, as described in the study by Summerfelt et al. [6] (package 4), increased the total number of eligible patients from 362 to 483 (63%). The highest number of eligible patients that could have received hospital-at-home care was found with package 5, which is the most elaborate care package.

### 3.2. Analysis of Package 3

The patients that could have received care at home with package 3 were younger (hospital median age of 65 (IQR 52–74) vs. home median age of 56 (IQR 42–68)), and the admission specialties and diagnoses were more diffusely divided (Table 4). These patients were also less sick, with a shorter length of stay and a lower mortality rate. The MEWS at presentation, however, was comparable in both groups. Of the care components of package 3 that were needed by the at-home care group, IV medication was the most frequent within 24 h (78%), and laboratory tests were most frequent in the following days (70% and 61% for 24–72 h and >72 h, respectively) (Table 5). After breaking down IV administration by type of medication, we found that most patients received intravenous antibiotics. The use of radiological imaging was diffusely spread over the periods of time. CT/MRI imaging was performed most often, although absolute numbers were small.

### 3.3. Identification of Eligibility Factors for Package 3

As the final part of our analysis, we assessed which factors could predict whether a patient would be eligible (or not eligible) for hospital-at-home care with the most feasible package: package 3. Older age, a higher MEWS, arriving by ambulance, and admission to a general internal medicine ward were factors that decreased the likelihood of being eligible for remote hospital care with package 3 at any point in time (Supplemental Table S4). For hospital-at-home care directly after an ED visit, being diagnosed with a lower respiratory tract infection was an additional factor that decreased the chance of being eligible. These factors did not impact eligibility for remote hospital care after 72 h. After 72 h, the chances of being eligible were higher for patients admitted to the nephrology department and patients with skin or viral infections. However, confidence intervals for these groups were wide, since only a few patients had one of these diagnoses.

## 4. Discussion

In this study, we explored which components of care are needed for a telemedicine-based hospital-at-home intervention for patients with infectious diseases. We found that the potential for hospital-at-home is high, and for one in three patients, it might already be possible directly after an ED visit. An intervention consisting of telemonitoring, virtual consultations, laboratory and radiologic diagnostics, medication, and oxygen therapy could potentially allow hospital-at-home care to almost half of our study population, regardless of admission diagnosis.

### 4.1. Selection of Care Components

The four components of hospital care in our study that were most frequently administered in all patient groups and time periods were ID/SC/IM medication, IV/other invasive medication, laboratory testing, and intercollegiate consultation. These are components that have also been frequently organized in previous hospital-at-home interventions [17,18,19,20,21]. Previously, components such as a urine catheter, central venous catheter, or feeding tube have been less frequently used. These care components require regular episodes of professional nursing care and are often a sign of more severe illness. Since oxygen therapy at home can easily be used by patients with relatively little guidance, we have added this component to the majority of care packages. Assistance in ADL is a common component of other hospital-at-home interventions; however, we have purposely left this out of the majority of packages, since assistance in ADL is one of the factors that makes hospital-at-home labor-intensive [22]. Although vital instability was uncommon among patients eligible for hospital care at home, an MEWS of 3 or higher still occurred, which is associated with a 12.7% chance of ICU admission [23]. The telemonitoring of vital signs therefore needs to be a basic component of virtual hospital care at home to allow for the early recognition of patient deterioration that may necessitate hospital re-admission. Regardless of the theoretical simplicity of organizing care components at home, it is important to keep in mind the reason why patients require certain care. The underlying disease, or especially uncertainty about the underlying disease, might be a reason to opt for hospitalization instead of hospital-at-home care.

### 4.2. Timing of Care

In this study, we found that a substantial number of patients were eligible for hospital-at-home care immediately following an ED visit and would therefore potentially not have to be admitted to the hospital at all. This type of hospital-at-home care is called ‘avoided admission’, as opposed to early ‘supported discharge’. Avoided admission by hospital-at-home care has been shown to provide benefits in clinical outcomes and costs [3]. Arranging the logistics, however, is more challenging, since all care needs to be organized during the relatively short ED visit. Admission-avoidance hospital-at-home care could even be taken further, by not sending the patient to the ED at all [24]. However, the majority of patients in our study received care in the ED, especially diagnostics—including imaging studies—and IV medication. A hospital-at-home intervention that also aims to avoid the ED visit will have to include a ‘fast track’ organization of these components at home, or at a minimum, the possibility of performing these at a primary care facility.

A finding of this study that might aid in early timing of the intervention is that the most frequently administered care components were fairly consistent when divided by admission diagnosis. This creates the possibility for a design that can be used regardless of admission diagnosis. If the design was to be disease specific, initiation of the intervention might in some cases have to be postponed until the diagnostical process is finished, which sometimes takes days and might therefore delay the transition of hospital care to the home situation. Furthermore, a design that is not dependent on diagnosis might also be of use for infectious diseases that are currently non-existing or not endemic. Lastly, one single design for all patients might be less expensive. We will elaborate on cost considerations in Section 4.4.

### 4.3. Patient Selection

Although hospital-at-home care is desirable, especially for elderly patients [4], a higher age was associated with less eligibility for hospital-at-home care with the selected package in our study. Elderly patients need more care, specifically care components that require frequent home visits such as ADL assistance. If the goal is to provide hospital-at-home care for elderly patients, home visits should not be avoided. Nonetheless, reasons for participating in hospital-at-home care might be even more patient-specific than we were able to investigate in this study. The availability and capacity of a supporting caregiver, the home situation, and the patient’s self-sufficiency have shown to be important factors in patient eligibility [25]. For hospital-at-home care supported by telemedicine specifically, the ability of a patient to work with telemonitoring and video consultation devices is crucial. This also requires more of a patient’s self-sufficiency than in-person hospital-at-home since many tasks will shift from the nurses’ responsibility to the patient’s responsibility. Furthermore, a subset of eligible patients will refuse hospital-at-home care, mainly because they prefer to receive care within the hospital, or have concerns about the safety of hospital-at-home care [20,26]. Since we have not measured these components in our study, the ultimate number of hospital-at-home patients will likely be smaller than the theoretical numbers reported in this study.

### 4.4. Cost Considerations

Although the use of telemedicine reduces the number of home visits, it does not necessarily reduce costs. A recent study on the use of telemonitoring for the early discharge of surgical patients found that the intervention was only cost-effective if implemented in all hospital wards, even without extra interventions such as diagnostics or medicinal treatment at home [8]. The reasons for this are the high costs for the purchase and maintenance of technology and the requirement of 24/7 staff availability. This intervention did not offer care at home besides monitoring and video consultation. Adding care components will increase costs but also increase the number of eligible patients. Moreover, valuable components including home visits are required to provide care for specific target populations, such as frail, elderly individuals or patients that need frequent clinical evaluation. The goal should therefore not be to avoid costly components but to design the most efficient intervention.

The organizational aspects of individual care components should also be taken into account if costs are to be assessed. A care component might cost more or less depending on the location at which this care is delivered. For example, radiological imaging to diagnose pneumonia could be performed in three ways. The first option is for the patient to be brought to the hospital by ambulance for a chest X-ray, and it costs EUR 760 for ambulance transport in the Netherlands [8]. Secondly, the patient could be brought by a family member or a taxi service, which costs significantly less. Lastly, the hospital could equip a professional with a portable ultrasound device and let the professional visit the patient to image the lungs, which is a valid alternative [27]. This will cost EUR 130 per visit [8], plus any costs for the purchase and maintenance of the device. An ultrasound probe might even be sent to the patient, who then images him/herself, guided by an expert over videoconferencing, and sends the images to the hospital, eliminating the need for the imaging visit altogether [28]. As seen in this example, the costs for hospital-at-home with telemonitoring are not easily calculated and will depend to a large extent on organizational choices.

### 4.5. Strengths and Limitations

In this study, we structurally investigated what types of care are administered during hospitalization to determine which of these might be offered at home. This is a first step towards hospital-at-home care based on the needs of the patient, rather than what the hospital can offer. We used a large cohort, representative of a complete year of admissions for various infectious diseases, and took various aspects of hospital care into consideration. Throughout the study, we made decisions driven by data, without losing sight of clinical relevance. This study also has several limitations. Due to the retrospective nature of the study, the decision-making process followed by hospital professionals that resulted in the patient being admitted was hard to determine. We might have missed components of care that always require hospital admission, which might have led to an overestimation of the proportion of patients with infectious diseases who are eligible for hospital-at-home care. Although we tried to take the frailty of patients into account by including care components such as physiotherapy consultation and ADL assistance, this is not equivalent to a formal frailty screening. Secondly, the study was conducted in a tertiary center with an accompanying specific patient population, and only for those patients who presented with a suspected infection. Conclusions can therefore only be generalized to similar populations. Some interventions to limit hospital admission were already in place during this study. For oncology patients, for example, the MASCC risk index [29] has been used since 2014 to determine whether a patient should be admitted with intravenous antibiotics or can be discharged with an oral variant, resulting in a preselected population. Lastly, we assumed that every component of care a patient received during admission was strictly necessary, which might not always be true. Previous studies have shown that patients with at-home hospital care typically receive fewer interventions than hospitalized patients, with similar patient outcomes [30,31]. The in-hospital overutilization of care components such as laboratory tests is well known [32]. The care received in the hospital might therefore not necessarily reflect the care needed at home. These limitations might have resulted in an underestimation of the population eligible for hospital-at-home care.

### 4.6. Future Perspectives

This study was limited by its retrospective design. In a prospective design, four questions in particular would be interesting to investigate: 1. What care do patients receive that is not recorded in the electronic health record?; 2. What part of the provided care is not strictly necessary and could be omitted?; 3. What are the reasons for hospital professionals to admit, or not yet discharge, a patient?; and 4. Of all patients that are theoretically eligible for hospital-at-home care, which patients are not able to, or do not wish to, participate and why? Answering these questions will further complete our understanding of the potential and optimal design of hospital-at-home care interventions for this population. Furthermore, a prospective study of the proposed intervention with care package 3 should be performed to test its feasibility, verify the number of patients that are eligible for intervention, and find components of care that are missing in this intervention. In this stage, all stakeholders should be involved, including patients, care-at-home providers, and general practitioners. In this study, cost-effectiveness and organizational impact should also be taken into consideration.

## 5. Conclusions

For patients currently admitted with a suspected infection, the potential for virtual hospital-at-home care is high. The proportion of eligible patients depends to a large extent on the required care components in the home setting. With a combination of telemonitoring and video consultation; laboratory and radiologic diagnostics; medication; and oxygen therapy, hospital-at-home care could potentially be offered to almost half of our population with suspected or proven infection. Certain factors are discernable at presentation that might predict eligibility for hospital-at-home care at a later stage. Our findings should be further investigated and validated prospectively to assess the validity and practical feasibility of the proposed intervention design, as well as costs and organizational aspects.

## Figures and Tables

**Table 1:** Patient characteristics.

	*N* = 763		*N* = 763
Age (median, IQR)	62 (47–72)	Admission specialty	
≥65	348 (46%)	- General Internal Medicine	263 (35%)
Female sex	360 (47%)	- Oncology	163 (21%)
CCI (median, IQR)	5 (2–7)	- Nephrology	124 (16%)
Medical history		- Haematology	112 (15%)
- Hypertension	385 (51%)	- Other	101 (13%)
- Diabetes	197 (26%)	Admission diagnosis	
- Chronic kidney disease	149 (20%)	- Lower respiratory tract infection	206 (27%)
Haemodialysis dependent	28 (4%)	- Urinary tract infection	141 (19%)
- Metastatic malignancy	140 (18%)	- Gastrointestinal infection	134 (18%)
- Haematological malignancy	100 (13%)	- Systemic viral infection	93 (12%)
- COPD	71 (9%)	- Skin infection	56 (7%)
Presented by ambulance	147 (19%)	- Non-infectious diagnosis	50 (7%)
MEWS at presentation (median, IQR)	3 (1–5)	- Unknown	44 (6%)
Length of hospitalization (median, IQR)	4 days (2–8)	- Other	39 (5%)
ICU admission during hospitalization	86 (11%)		
Died during hospitalization	46 (6%)		

IQR: interquartile range, CCI: Charlson Comorbidity Index, MEWS: Modified Early Warning Score, ICU: intensive care unit.

**Table 2:** Percentage of admitted patients receiving care components during four stages of admission.

	ED *N* = 763	<24 h *N* = 763	24 h–72 h *N* = 705	>72 h *N* = 496
Diagnostics				
- Laboratory	758 (99%)	498 (65%)	566 (80%)	405 (82%)
- X-ray/U	603 (79%)	134 (18%)	130 (18%)	170 (34%)
- CT/MRI/other *	120 (16%)	95 (13%)	63 (9%)	102 (21%)
Interventions				
- Oxygen therapy 1–5 L/min	123 (16%)	208 (27%)	155 (22%)	119 (24%)
- Oxygen therapy > 5 L/min	43 (6%)	43 (6%)	21 (3%)	28 (6%)
- ID/SC/IM medication	317 (42%)	345 (45%)	334 (47%)	265 (53%)
- IV/other hospital medication ^	659 (86%)	629 (82%)	540 (77%)	322 (65%)
- Central intravenous catheter	11 (1%)	6 (0.8%)	9 (1%)	19 (4%)
- Urine catheter	28 (4%)	158 (21%)	163 (23%)	126 (25%)
- Feeding tube	3 (0.4%)	67 (9%)	72 (18%)	82 (17%)
- High care intervention †	14 (2%)	33 (4%)	31 (4%)	51 (10%)
- Intercollegiate consultation	50 (7%)	428 (56%)	420 (60%)	342 (69%)
- RRT consultation	4 (0.5%)	12 (2%)	4 (0.6%)	4 (0.8%)
- ICU admission	55 (7%)	62 (8%)	64 (9%)	44 (9%)
Patient stability and self-reliance				
- MEWS ≥ 3	ND	208 (27%)	151 (21%)	129 (26%)
- MEWS ≥ 5	ND	112 (15%)	72 (10%)	72 (15%)
- Assistance in ADL	ND	138 (18%)	148 (21%)	173 (35%)
- Physiotherapist consultation	0 (0%)	30 (4%)	122 (17%)	209 (42%)
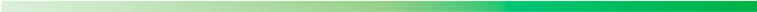 0% of patients 100% of patients				

ED: emergency department, ID: intradermal, SC: subcutaneous, IM: intramuscular, IV: intravenous/other invasive, RRT: rapid response team, MEWS: Modified Early Warning Score, ADL: Activities of Daily Living, IQR: interquartile range, ND: no data. * Other imaging: PET/CT, lung perfusion and/or ventilation scan. ^ Other hospital medication: medication administration for which additional care and/or expertise is needed, such as peritoneal or intravesicular administration, or medication via feeding tube. † High care intervention: surgery, bronchoscopy, cystoscopy, endoscopy, transoesophageal ultrasound, cardioversion, radiologic intervention, peripheral nerve block, and similar procedures.

**Table 3:** Care package compositions.

**Package**	**Rationale**	**Care Components**
Basic package	Components that can always be arranged for every patient. Intradermal, subcutaneous and/or intramuscular medication administration can be taught to the patient and/or a caregiver.	· Telemonitoring and video consultation · Intercollegiate consultation · Intradermal/subcutaneous/intramuscular medication
1.	The most important problem is the self-sustainability of the patient. The patient cannot easily come to a clinic.	Basic package + · Laboratory tests · ADL assistance · Physiotherapy
2.	Simple diagnostics and therapy that a patient can receive without assistance. The patient is mobile and can go to an outpatient clinic for diagnostics if necessary.	Basic package + · Laboratory tests · Radiology tests · Oxygen therapy
3.	Slightly more elaborate diagnostics; a trained professional is needed for IV medication.	Package 2 + · Intravenous/other invasive medication
4. (Summerfelt)	The study by Summerfelt et al. reports an existing hospital-at-home intervention in which in-person visits were replaced with video consultation and telemonitoring as much as possible.	Package 3 + · ADL assistance · Physiotherapy
5.	The majority of components can be arranged at home; however, there is a limit on the number of certain components, so as to not overburden home healthcare professionals.	Package 4 + · Urine catheter OR central venous catheter OR feeding tube

**Table 4:** Characteristics of patients eligible for hospital-at-home care with package 3.

	>ED		>24 h		>72 h	
	Hospital (*N* = 499)	Home (*N* = 264)	Hospital (*N* = 433)	Home (*N* = 272)	Hospital (*N* = 328)	Home (*N* = 168)
Characteristics						
- Age (median, IQR)	65 (52–74)	56 (42–68)	66 (52–74)	58 (45–70)	65.5 (52–74)	61.5 (46–72)
- CCI (median, IQR)	5 (3–7)	4 (2–6)	5 (3–7)	4 (2–6)	5 (3–7)	4 (2–7)
- Admission specialty						
· General Internal Medicine	211 (42%)	59 (22%)	189 (44%)	57 (21%)	140 (43%)	41 (24%)
· Oncology	116 (23%)	47 (18%)	97 (22%)	53 (20%)	74 (23%)	29 (17%)
· Nephrology	64 (13%)	60 (23%)	56 (22%)	61 (22%)	40 (12%)	49 (29%)
· Hematology	56 (11%)	56 (21%)	45 (10%)	62 (23%)	39 (12%)	31 (19%)
· Other	52 (10%)	42 (16%)	46 (11%)	39 (14%)	35 (11%)	18 (11%)
- Admission diagnosis						
· Lower respiratory tract infection	163 (33%)	43 (16%)	142 (33%)	52 (19%)	109 (33%)	39 (23%)
· Urinary tract infection	101 (20%)	40 (15%)	87 (20%)	49 (18%)	61 (19%)	32 (19%)
· Gastrointestinal tract infection	77 (15%)	57 (22%)	63 (15%)	58 (21%)	46 (14%)	32 (19%)
· Viral infection	41 (8%)	52 (20%)	34 (8%)	47 (17%)	23 (7%)	28 (17%)
· Skin infection	31 (6%)	25 (10%)	26 (6%)	26 (10%)	21 (6%)	14 (8%)
· Other	86 (17%)	47 (18%)	81 (19%)	40 (15%)	68 (21%)	23 (14%)
- Length of hospitalization (median, IQR)	6 (4–10)	2 (2–4)	7 (4–11)	3 (2–4)	9 (6–13)	4 (4–6)
- Died (n, %)	43 (9%)	3 (1%)	36 (8%)	2 (0.7%)	2 (0.6%)	1 (0.6%)
- MEWS ≥ 3	167 (34%)	41 (16%)	184 (43%)	21 (78%)	118 (36%)	11 (7%)
- MEWS ≥ 5	100 (20%)	12 (5%)	108 (25%)	4 (2%)	69 (21%)	3 (2%)
- MEWS at presentation (median, IQR)	3 (1–5)	2 (1–4)	3 (2–5)	2 (1–4)	3 (2–5)	2 (1–4)

ED: emergency department, IQR: interquartile range, MEWS: Modified Early Warning Score.

**Table 5:** Percentage of patients eligible for hospital-at-home care with package 3 receiving care components.

	**<24 (*N* = 264)**	**24–72 h (*N* = 272)**	**>72 h (*N* = 168)**
Diagnostics			
- Laboratory test	143 (54%)	190 (70%)	103 (61%)
- Radiological imaging	33 (13%)	29 (11%)	21 (13%)
· X-ray	6 (2%)	10 (4%)	12 (7%)
· Ultrasound	17 (6%)	8 (3%)	0 (0%)
· CT/MRI	13 (5%)	15 (6%)	8 (5%)
· Other medical imaging	0 (0%)	0 (0%)	3 (2%)
- Intercollegiate consultation	115 (44%)	114 (42%)	72 (43%)
Interventions			
- Oxygen therapy 1–5 L/min	32 (12%)	20 (7%)	7 (4%)
- ID/SC/IM medication	88 (33%)	92 (34%)	58 (35%)
- IV/other hospital medication	205 (78%)	189 (70%)	82 (49%)
· Resuscitation fluid	12 (5%)	9 (3%)	7 (4%)
· Blood products	1 (0.4%)	1 (0.4%)	1 (0.6%)
· Antibiotics	166 (63%)	162 (60%)	65 (39%)
· Else	85 (32%)	74 (27%)	37 (22%)
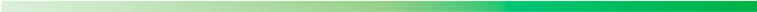 0% of patients 100% of patients			

## Data Availability

Due to ongoing research with the data used in this study, the data are currently not available.

## Supplementary Materials

The following supporting information can be downloaded at: https://www.mdpi.com/article/10.3390/jcm13040977/s1, Table S1: Percentage of admitted patients receiving care components during four periods of admission for patients with lower respiratory tract infections. Table S2: Percentage of admitted patients receiving care components during four periods of admission for patients with urinary tract infections. Table S3: Percentage of admitted patients receiving care components during four periods of admission for patients with gastrointestinal tract infections. Table S4: Prediction of eligibility for hospital-at-home care with package 3.
