# Supplementary material for: Designing a Virtual Hospital-at-Home Intervention for Patients with Infectious Diseases: A Data-Driven Approach
Source: J Clin Med. 2024 Feb 8;13(4):977. doi: 10.3390/jcm13040977 (PMC10889708; doi:10.3390/jcm13040977)
Supplement: Supplementary file 1 [file jcm-13-00977-s001.zip › Supplemental table S1.pdf]

Supplemental table S1. Percentage of admitted patients receiving care components during four periods of admission for patients with lower respiratory tract infections

	ED<br>N=206	<24h<br>N=206	24h-72h<br>N=194	>72h<br>N=148
Diagnostics				
- lab	206 (100%)	137 (67%)	158 (81%)	119 (80%)
- X-ray/U	197 (96%)	41 (20%)	45 (23%)	61 (41%)
- CT/MRI/other	35 (17%)	93 (45%)	15 (8%)	32 (22%)
Interventions				
- Oxygen therapy 1-5L/min	68 (33%)	108 (52%)	87 (45%)	59 (40%)
- Oxygen therapy >5L/min	23 (11%)	29 (14%)	16 (8%)	15 (10%)
- ID/SC/IM medication	93 (45%)	98 (48%)	93 (48%)	80 (54%)
- IV / other hospital medication^	183 (89%)	181 (88%)	140 (72%)	87 (59%)
- Central intravenous catheter	5 (3%)	1 (0.5%)	2 (1%)	4 (3%)
- Urine catheter	8 (4%)	49 (24%)	50 (26%)	40 (27%)
- Feeding tube	1 (0.5%)	29 (14%)	31 (16%)	29 (20%)
- High care intervention†	3 (2%)	8 (4%)	2 (1%)	10 (7%)
- Intercollegiate consultation	15 (7%)	116 (56%)	126 (65%)	112 (76%)
- RRT consultation	3 (2%)	6 (3%)	4 (2%)	2 (1%)
- ICU admission	21 (10%)	27 (13%)	27 (14%)	20 (14%)
Patient stability and self-reliance				
- MEWS ≥ 3	ND	89 (43%)	70 (36%)	54 (37%)
- MEWS ≥ 5	ND	57 (28%)	33 (17%)	35 (24%)
- Assistance in ADL	ND	44 (21%)	48 (25%)	61 (41%)
- Physiotherapist consultation	0 (0%)	12 (6%)	52 (27%)	84 (57%)

0% of patients — 100% of patients

ED: emergency department, ID: intradermal, SC: subcutaneous, IM: intramuscular, IV: intravenous/other invasive, RRT: rapid response team, MEWS: Modified Early Warning Score, ADL: Activities of Daily Living, IQR: interquartile range, ND: no data.

*Other imaging: PET/CT, lung perfusion and/or ventilation scan. ^Other hospital medication: medication administration for which additional care and/or expertise is needed, such as peritoneal or intravesicular administration, or medication via feeding tube. †High care intervention: surgery, bronchoscopy, cystoscopy, endoscopy, transesophageal ultrasound, cardioversion, radiologic intervention, peripheral nerve block, and similar procedures.
