# Supplementary material for: Designing a Virtual Hospital-at-Home Intervention for Patients with Infectious Diseases: A Data-Driven Approach
Source: J Clin Med. 2024 Feb 8;13(4):977. doi: 10.3390/jcm13040977 (PMC10889708; doi:10.3390/jcm13040977)
Supplement: Supplementary file 1 [file jcm-13-00977-s001.zip › Supplemental table S2.pdf]

Supplemental table S2. Percentage of admitted patients receiving care components during four periods of admission for patients with urinary tract infections

	ED N=141	<24h N=141	24h-72h N=136	>72h N=93
Diagnostics				
- lab	140 (99%)	96 (68%)	116 (85%)	76 (82%)
- X-ray/U	107 (76%)	23 (16%)	21 (15%)	26 (28%)
- CT/MRI/other	15 (11%)	15 (11%)	13 (10%)	12 (13%)
Interventions				
- Oxygen therapy 1-5L/min	19 (14%)	28 (20%)	21 (15%)	14 (15%)
- Oxygen therapy >5L/min	9 (6%)	5 (4%)	0 (0%)	2 (2%)
- ID/SC/IM medication	62 (44%)	70 (50%)	69 (51%)	51 (55%)
- IV / other hospital medication^	129 (92%)	122 (87%)	115 (85%)	59 (63%)
- Central intravenous catheter	3 (2%)	0 (0%)	1 (0.7%)	2 (2%)
- Urine catheter	11 (8%)	45 (32%)	50 (37%)	37 (40%)
- Feeding tube	1 (0.7%)	6 (4%)	5 (4%)	6 (7%)
- High care intervention†	5 (4%)	6 (4%)	7 (5%)	8 (9%)
- Intercollegiate consultation	5 (4%)	77 (55%)	73 (54%)	57 (61%)
- RRT consultation	0 (0%)	2 (1%)	0 (0%)	1 (1%)
- ICU admission	11 (8%)	12 (9%)	11 (8%)	7 (8%)
Patient stability and self-reliance				
- MEWS ≥ 3	ND	34 (24%)	23 (17%)	22 (24%)
- MEWS ≥ 5	ND	17 (12%)	10 (7%)	8 (9%)
- Assistance in ADL	ND	29 (21%)	33 (24%)	29 (31%)
- Physiotherapist consultation	0 (0%)	4 (3%)	27 (20%)	31 (33%)

0% of patients — 100% of patients

ED: emergency department, ID: intradermal, SC: subcutaneous, IM: intramuscular, IV: intravenous/other invasive, RRT: rapid response team, MEWS: Modified Early Warning Score, ADL: Activities of Daily Living, IQR: interquartile range, ND: no data.

*Other imaging: PET/CT, lung perfusion and/or ventilation scan. ^Other hospital medication: medication administration for which additional care and/or expertise is needed, such as peritoneal or intravesicular administration, or medication via feeding tube. †High care intervention: surgery, bronchoscopy, cystoscopy, endoscopy, transesophageal ultrasound, cardioversion, radiologic intervention, peripheral nerve block, and similar procedures.
