# Supplementary material for: Designing a Virtual Hospital-at-Home Intervention for Patients with Infectious Diseases: A Data-Driven Approach
Source: J Clin Med. 2024 Feb 8;13(4):977. doi: 10.3390/jcm13040977 (PMC10889708; doi:10.3390/jcm13040977)
Supplement: Supplementary file 1 [file jcm-13-00977-s001.zip › Supplemental table S3.pdf]

Supplemental table S3. Percentage of admitted patients receiving care components during four periods of admission for patients with gastrointestinal infections

	ED<br>N=134	<24h<br>N=134	24h-72h<br>N=121	>72h<br>N=78
Diagnostics				
- lab	134 (100%)	98 (73%)	93 (77%)	66 (85%)
- X-ray/U	75 (56%)	26 (19%)	12 (10%)	20 (26%)
- CT/MRI/other	28 (21%)	15 (11%)	10 (8%)	15 (19%)
Interventions				
- Oxygen therapy 1-5L/min	10 (8%)	19 (14%)	15 (12%)	15 (19%)
- Oxygen therapy >5L/min	3 (2%)	3 (2%)	2 (2%)	4 (5%)
- ID/SC/IM medication	55 (41%)	62 (46%)	60 (50%)	44 (56%)
- IV / other hospital medication^	112 (84%)	101 (75%)	86 (71%)	59 (76%)
- Central intravenous catheter	1 (0.7%)	2 (2%)	1 (0.8%)	3 (40%)
- Urine catheter	2 (2%)	24 (18%)	17 (14%)	12 (15%)
- Feeding tube	1 (0.7%)	6 (5%)	13 (11%)	14 (18%)
- High care intervention†	1 (0.7%)	12 (9%)	9 (7%)	13 (17%)
- Intercollegiate consultation	11 (8%)	77 (58%)	67 (55%)	54 (69%)
- RRT consultation	0 (0%)	2 (2%)	0 (0%)	1 (1%)
- ICU admission	6 (5%)	7 (5%)	7 (6%)	4 (5%)
Patient stability and self-reliance				
- MEWS ≥ 3	ND	56 (19%)	17 (14%)	15 (19%)
- MEWS ≥ 5	ND	12 (9%)	10 (8%)	10 (13%)
- Assistance in ADL	ND	27 (20%)	20 (17%)	28 (36%)
- Physiotherapist consultation	0 (0%)	4 (3%)	11 (9%)	23 (30%)

0% of patients — 100% of patients

ED: emergency department, ID: intradermal, SC: subcutaneous, IM: intramuscular, IV: intravenous/other invasive, RRT: rapid response team, MEWS: Modified Early Warning Score, ADL: Activities of Daily Living, IQR: interquartile range, ND: no data.

*Other imaging: PET/CT, lung perfusion and/or ventilation scan. ^Other hospital medication: medication administration for which additional care and/or expertise is needed, such as peritoneal or intravesicular administration, or medication via feeding tube. †High care intervention: surgery, bronchoscopy, cystoscopy, endoscopy, transesophageal ultrasound, cardioversion, radiologic intervention, peripheral nerve block, and similar procedures.
