# Supplementary material for: Designing a Virtual Hospital-at-Home Intervention for Patients with Infectious Diseases: A Data-Driven Approach
Source: J Clin Med. 2024 Feb 8;13(4):977. doi: 10.3390/jcm13040977 (PMC10889708; doi:10.3390/jcm13040977)
Supplement: Supplementary file 1 [file jcm-13-00977-s001.zip › Supplemental table S4.pdf]

Supplemental table S4. Prediction of eligibility for hospital-at-home care with package 3

		>any point		>ED		>24h		>72h	
		OR	95%CI	OR	95%CI	OR	95%CI	OR	95%CI
Age		**0.99**	**0.98-1.00**	**0.98**	**0.97-0.99**	**0.99**	**0.98-0.99**	-	
MEWS at ED		**0.91**	**0.84-0.98**	**0.88**	**0.81-0.95**	**0.90**	**0.83-0.98**	0.93	0.84-1.02
Brought in by ambulance		**0.50**	**0.32-0.78**	**0.41**	**0.24-0.70**	**0.44**	**0.26-0.73**	-	
Admission specialty*	General Internal Medicine	**0.46**	**0.28-0.78**	**0.50**	**0.29-0.86**	**0.45**	**0.26-0.78**	0.62	0.31-1.24
	Oncology	0.67	0.39-1.15	0.58	0.33-1.02	0.69	0.39-1.22	0.78	0.38-1.63
	Nephrology	1.44	0.80-2.61	1.34	0.74-2.44	1.24	0.68-2.28	**2.60**	**1.22-5.52**
	Hematology	1.18	0.65-2.15	1.37	0.75-2.51	1.72	0.93-3.20	1.42	0.65-3.07
Admission diagnosis^	LRTI	0.81	0.39-1.71	**0.41**	**0.19-0.89**	0.61	0.27-1.35	2.42	0.64-9.01
	UTI	0.82	0.38-1.77	0.48	0.21-1.07	0.71	0.35-1.84	2.38	0.62-9.24
	GI	1.29	0.60-2.78	0.75	0.34-1.64	0.61	0.27-1.35	3.68	0.94-14.4
	Skin infection	1.80	0.75-4.32	1.10	0.45-2.68	1.69	0.66-4.31	**4.88**	**1.13-21.1**
	Viral infection	2.19	0.97-4.94	1.25	0.55-2.82	1.45	0.61-3.43	**6.07**	**1.50-24.6**
	Other diagnosis	0.84	0.38-1.88	0.53	0.23-1.23	0.50	0.21-1.20	2.55	0.64-10.2

OR: odds ratio, CI: confidence interval, ED: emergency department, MEWS: Modified Early Warning Score, LRTI: lower respiratory tract infection, UTI: urinary tract infection, GI: gastrointestinal infection.

*Reference category: other specialties. ^Reference category: unknown diagnosis.
